# Monitoring of Laboratory Reared of *Phlebotomus papatasi* (Diptera: Psychodidae), Main Vector of Zoonotic Cutaneous Leishmaniasis to Different Imagicides in Hyper endemic Areas, Esfahan Province, Iran

**DOI:** 10.18502/jad.v14i1.2718

**Published:** 2020-03-31

**Authors:** Leila Shirani-Bidabadi, Ali Reza Zahraei-Ramazani, Mohammad Reza Yaghoobi-Ershadi, Amir Ahmad Akhavan, Mohammad Ali Oshaghi, Ahmad Ali Enayati, Yavar Rassi, Fatemeh Gholampour, Niloufar Shareghi, Elham Madreseh, Hassan Vatandoost

**Affiliations:** 1 Department of Vector Biology and Control, School of Health, Kerman University of Medical Sciences, Kerman, Iran; 2 Department of Medical Entomology and Vector control,School of Public Health, Tehran University of Medical Sciences, Tehran, Iran; 3 Department of Medical Entomology and Vector control, School of Public Health, Mazandaran University of Medical Sciences, Mazandaran, Iran; 4 Esfahan Health Research Station, National Institute of Health Research ,Tehran University of Medical Sciences, Tehran, Iran; 5 Department of Epidemiology and Biostatistics, School of Public Health, Tehran University of Medical Sciences, Tehran, Iran; 6 Department of Chemical Polutants and Pesticides, Institute for Environmenatl Research, Tehran University of Medical Sciences, Tehran, Iran

**Keywords:** *Phlebotomus papatasi*, Bioassay, Insecticide resistance, Rearing

## Abstract

**Background:**

In domestic and per domestic area, insecticides such as DDT, malathion, fenitrothion, propoxur and, more recently, synthetic pyrethroids such as deltamethrin and lambda-cyhalothrin, have been successfully used to control sand flies in many countries. The present study reports the results of time-mortality bioassay to DDT 4%, lambda-cyhalothrin 0.05%, permethrin 0.75%, cyfluthrin 0.15% and deltamethrin 0.05% in recently colonized *Phlebotomus papatasi* populations in Iran.

**Methods:**

The insecticide susceptibility status of *P. papatasi* laboratory population was assessed during 2016–2017, following the standard WHO technique for mosquito (WHO, 2013) based on diagnostic dose. Sand flies collected from rural area of Badrood (Matin Abad), Natanz County, Esfahan Province, using aspirator.

**Results:**

Susceptibility test to DDT and pyrethroids was assessed on 3534 laboratory-reared *P. papatasi* (1746 females and 1788 males). The LT _50_ and LT _90_ values were measured using probit analysis and regression lines. The test results against males of *P. papatasi* revealed that LT _50_ values to DDT 4%, Permethrin 0.75%, Deltamethrin 0.05%, Cyfluthrin 0.15% and Lambdacyhalothrin 0.05% were 439.28, 108.90, 97.75, 5.00 and 57.84 seconds. The figures for females were 641.62, 136.15, 146.44, 8.71 and 72.69 seconds, respectively.

**Conclusion:**

According to presented results, the reared population of sand flies collected from a hyper-endemic region of Esfahan Province is still susceptible to prethroids and Resistance candidate to DDT 4%.

## Introduction

Phlebotomine sand flies (Diptera, Psychodidae) are hematophagous insects involved in the transmission of viruses (Bunyaviridae, Reoviridae and Rhabdoviridae), bacteria (Bartonella bacilliformis) and protozoa (Leishmania spp.) to animals and humans (1–3). Among protozoa, Leishmania spp. are recognized as pathogenic to humans, causing different clinical forms: visceral (VL), cutaneous (CL), mucocutaneous, post-kala-azar dermal and mucosal leishmaniasis (4). Leishmaniases are neglected diseases worldwide distributed, occurring mainly in tropical and subtropical zones (5). Cutaneous leishmaniasis (CL) is caused by *Leishmania major*, *L. tropica* or *L. infantum*, while VL is caused by *L. infantum* (6). There are 56 species (32 *Phlebotomus* and 24 *Sergentomyia*) of phlebotomine sandflies in Iran but *Phlebotomus papatasi* is the main vector of Zoonotic Cutaneous Leishmaniasis (ZCL) (7–10). Few studies have been performed on the level of susceptibility of sandflies reared in the laboratory to common insecticides used in agriculture and health in world. Hassan et al. (2012) in Sudan studied insecticide susceptibility status of first progeny (F1) of *P. papatasi* to DDT, permethrin, malathion, and propoxur. This study results showed sand flies were resistance to malathion and propoxur (11). In Iran sand flies *P. papatasi* which were collected from a hyper endemic focus of the disease in central Iran breed under laboratory condition and determine sand flies susceptibility level to commonly used insecticides (12). Denlinger et al. in 2016 showed that *P. papatasi* and *Lutzomyia longipalpis* sand flies are highly susceptible to the carbamates as their diagnostic doses are under 7.0μg/ml. Both species are also highly susceptible to DDT during the exposure assay as their diagnostic doses are 7.5μg/ml, yet their diagnostic doses for the 24h recovery period are 650.0μg/ml for *Lu. longipalpis* and 470.0 μg/ml for *P. papatasi* (13). Italian populations of *P. perniciosus* and *P. papatasi* from Campania region and from Rome, respectively, were susceptible to the insecticides DDT 2%, lambda-cyhalothrin 0.06% and permethrin 0.2% as compared with the reference strain used (14). The present study reports the results of time-mortality bioassay to DDT 4%, lambda-cyhalothrin 0.05%, permethrin 0.75%, cyfluthrin 0.15 % and deltamethrin 0.05% in recently colonized *P. papatasi* populations in Iran.

## Materials and Methods

### Study area

This study was performed during the summer of 2016, 2017 and sand flies were caught from the rural district (Matin Abad) of Badrood (33° 44′ N, 52° 2′ E), Natanz County, Esfahan Province, central of Iran. The area is located in the desert with hot and dry weather in summer and quite cold in winter. This zone is a Zoonotic Cutaneous Leishmaniasis (ZCL) focus in Iran. In this area were exsistence many farm lands. Crops such as wheat, sunflower, alfalfa, clover and other are planted in the area. Due to intensive use of insecticides for agriculture pests, the leishmania vectors are exposed to insecticide selection pressures.

### Collected sand flies from field

Sand flies were collected outdoor near their breeding places using hand aspirator device from sunset to midnight during the period of June to August 2016, 2017. Collected alive sand flies were released into a clean cage with a hanging piece of wet cloth for supplying suitable humidity and feeding on a 20% sucrose solution soaked cotton. Sand flies cage were placed in a plastic bag to remain wet and to keep stable temperature situation. The cages were transported to sand fly insectary in Esfahan Health Research Station, Tehran University of Medical Sciences. Appropriate condition for rearing and inbreeding of sand flies in laboratory were 25±2 °C and 72±9.6% relative humidity in insectary and 90±7% RH in rearing box (15). Temperatures were maintained by automated electric heaters and photoperiod of 14/10 D/L was maintained in the insectary.

### Sand fly rearing method

After resting sand flies in insectary, fed and gravid wild-caught female adults were separated by aspirator and were released into individual pots according to Volf and volfa method (16) and were fed with honey solution (50 %) and saturated sucrose. Engorged female were fed on Blab/C that anesthetized with 0.2 cc Ketamine and Xylazine for 30 minutes per mice. After feeding, blood-engorged sand flies were individually put in oviposition vials lined at the bottom with plaster of Paris and covered with mesh. The vials containing females were then maintained at 26–28 °C and 72±10% RH. After oviposition females died, then females were removed from oviposition vials and were preserved individually in 70% alchohol for mounting and identification up to the species level by using proper identification entomology keys (17). Females and males at least one week after mounting, were identified using valid key and if there were any other species, all of them were excluded from the tests. Males and Females of *P. papatasi* females were separated from other species for rearing. The pots were checked daily for hatching the eggs. The larvae (L1) were fed with larval food, complex of rabbit food (palette) and rabbit feces with liver powder (18). For mass rearing of sand flies used larger pots that linned with plaster of Paris, then we transferred 20–30 blood feeding females with 5–10 males. Emerged adults were released in a new cage with wet cloth and sucrose solution (20%). All of them were placed in a plastic bag to remain wet and to maintain stable temperature situation. Then the 3–10 days old adults were tested in a standard WHO susceptibility test method as described for mosquitoes (19).

### Problems with Sand fly breeding

We have many problems in mass rearing of sand flies in insectarium including: collection of sand flies from field because of wind in night and decrease temperature in midnight, decrease of density of sand flies due to interventions control of Esfahan Deputy of Health and Provincial Health Center in study areas in recent year, preparation of proper humidity and temperature in insectary for sand fly rearing, fungal contamination in all of stage, mites, cannibalism (especially in first larval stage) diapause of fourth larval stage. In caused occasional contamination of food with fungi belonging to genus *Aspergillus*, *Mucor* and *Cladosporium*, mites (Super Cohort: Monogynaspida), insects from the order Psocoptera and Ants were observed during any stage of rearing process (18). Our serious problems have been fungi such as *Mucor* sp., *Cladosporium* sp. and *Aspergillus* sp., bacteria such as *Pseudomonas* sp., *Salmonella* sp., *Diphteroide* sp. and mites (Order Mesostigmata).

### Susceptibility bioassays of reared sand flies

Susceptibility test against specimens reared in laboratory condition, were carried out in summer of 2016, 2017. The susceptibility tests were carried out on 3534 laboratory-reared *P. papatasi* (1746 females and 1788 males). WHO test-kit tubes and impregnated papers were procured from collaborating Center of WHO in Malaysia. All the susceptibility tests were done according to standard WHO testing protocols on unfed female sand flies using at least 8–15 specimens in per test. Emerged sand flies transferred to cages, after resting and feeding by sucrose solution (20%), sand flies were tested according to the standard method of WHO (19). The sand flies were transferred into the exposure tubes by aspirator and were gently transferred to the holding tube at different time intervals and then the mortality was counted after 24h recovery period. After exposure period sand flies fed with 20% sugar solution placed in the top of the holding tube. Control test tubes carrying control papers were also held parallel to each set of tests. All the tests were ignored if the mortality was higher than 20% in the control group. The test was done in four to six replicates for each insecticide. During these bioassays tests, laboratory condition was stabilized at (25±2 °C and 73±10% RH) in insectary. The resistance status of sand fly specimens was determined according to the latest WHO criteria (19) as follows, (a) mortality rates between 98–100% indicate full susceptibility, (b) mortality rates between 90–97% require further investigation, (c) mortality rates < 90 %, the population is considered resistant to the tested insecticides (19). After each test, all the dead and alive sand flies were transferred to 70% alcohol separately for subsequent study. The exposure time interval was between 7, 14, 28, 56, 113, 225, 450, 900, 1800 and 3600 seconds. At least 8–10 interval times were used to gain the mortality between 5 and 95%. In each exposure time at least 4–6 replicates were used comprising 50–100 sand flies depending on the availability and the same age of the adults. Abbott’s formula was not used to correct experimental mortalities if the control group mortality was <5%. If control group mortalities exceeded 20%, the entire testing replicate was not used (20).

### Insecticides papers used in bioassay test

Impregnated papers with different diagnostic dosages of DDT 4% (Batch number: DD 214), Permethrin 0.75% (Batch number: PE 340), Deltamethrin 0.05% (Batch number: DE 432), Lambda-cyhalothrin 0.05% (Batch number: LA215) and Cyfluthrin 0.15% (Batch number: CY098) were taken from collaborating center of WHO in Malaysia.

### Obtaining data and analyses

The exposure time versus probit mortality were used according to Finney 1971. The Excel 2013 was used for data entering. Data analysis was done with statistic software spss version 22. Excel software version 2013 was used for drawing the regression line.

## Results

### Identification of sand flies

All females and males specimens (2942 Number) used for the establishment of the colonies of sand flies from the Badrood population were identified by avalaible key morphologically as *P. papatasi*.

### Bioassay test of laboratory sand flies

**Fig. 1.**
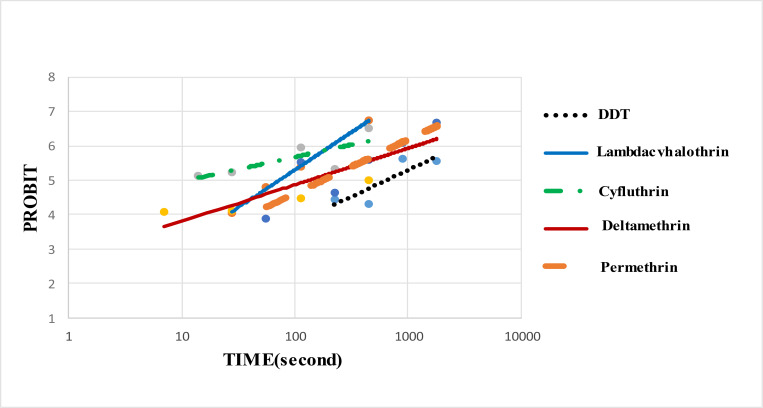
Probit regression lines of different insecticides against female of *Phlebotomus papatasi* laboratory population, Esfahan, Iran, 2016–2017

**Fig. 2.**
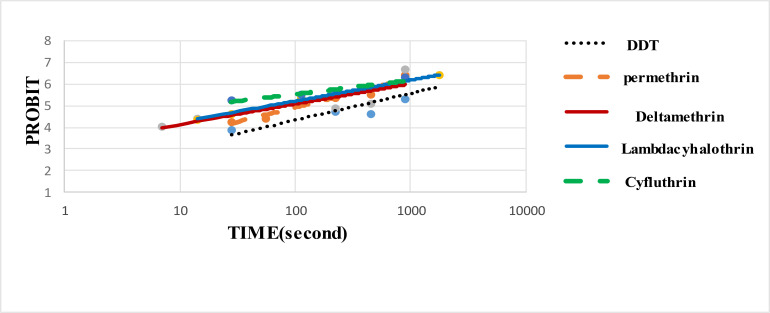
Probit regression lines of different insecticides against male of *Phlebotomus papatasi* laboratory population, Esfahan, Iran, 2016–2017

**Fig. 3.**
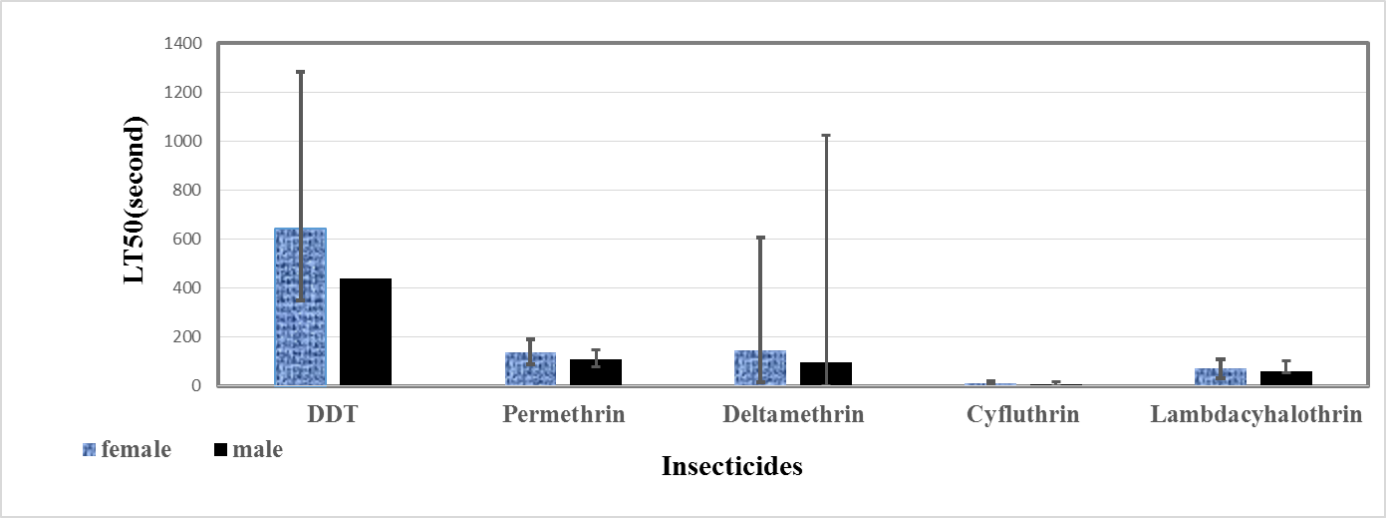
LT _50_ values of different insecticides against male and female of *Phlebotomus papatasi* laboratory populations, Esfahan, Iran, 2016–2017

In this study 2942 sand fly specimens collected from study area were reared in insectary. The results of susceptibility test against laboratory -reared *P. papatasi* female revealed LT50 values to DDT (4%), permethrin (0.75%), deltamethrin (0.05%), cyfluthrin (0.15%) and lambda-cyhalothrin (0.05%); 641.62, 136.15, 146.44, 8.71, 72.69 seconds, respectively (Table 1). This data for males to DDT (4%), permethrin (0.75%), deltamethrin (0.05%), cyfluthrin (0.15%) and lambda-cyhalothrin (0.05%) were 439.28, 108.90, 97.75, 5.00, 57.84 seconds respectively (Table 2). Figures 1, 2 show the probit regression lines. The results showed that males were more susceptible than females to all the insecticides tested at LT _50_ level (Fig. 3). The results showed that males were more susceptible than females to all the insecticides tested at LT _50_ level (Fig. 3).

**Table 1.** Parameters of probit regression lines of different insecticides against female *Phlebotomus papatasi*, laboratory strain, Esfahan, Iran, 2016–2017

**Insecticide**	**A^a^**	**B±SE^b^**	**LT_50_^c^ 95%CL (seconds)**	**LT_90_^d^ 95%CL (seconds)**	**Χ^2^ (df)**	**Heterogeneity P-value**	**Y=a+bx**
**DDT 4%**	−4.3272	1.5414±0.514	350.23	1822.01	3.281 (2)	P> 0.05	Y= −4.3272+ 1.5414 X
			641.62	4352.28			
			1283.51	196819.15			
**Permethrin 0.75%**	−2.8509	1.9533±0.499	87.63	778.65	25.164 (4)	P< 0.05	Y= −2.8509+ 1.3359 X
			136.15	1239.85			
			189.47	2667.51			
**Deltamethrin 0.1%**	−2.1764	1.0049±0.259	13.82	651.23	13.600 (4)	P< 0.05	Y= −2.1764+ 1.0049 X
			146.44	2760.47			
			604.09	2254569.23			
**Cyfluthrin 0.15%**	−0.5400	0.5743±0.114	2.50	434.62	9.183 (5)	P> 0.05	Y= −0.5400+ 0.5743 X
			8.71	1485.06			
			18.69	19486.52			
**Lambdacyhalothrin 0.05%**	−4.0834	2.6391±0.410	52.59	176.75	0.091 (2)	P> 0.05	Y= −4.0834+ 2.1936 X
			72.69	279.09			
			101.65	675.84			

a A= Intercept

b B±SE= Slope and its Standard Error

c LT_50_, 95% C.L.= Leathal time cause 50% mortality and its 95% Confidence Limits

d LT_90_, 95% C.L.= Leathal time cause 90% mortality and its 95% Confidence Limits

**Table 2.** Parameters of probit regression lines of different insecticides against male *Phlebotomus papatasi* laboratory population, Esfahan, Iran, 2016–2017

**Insecticide**	**A^a^**	**B±SE^b^**	**LT_50_^c^ 95% C.L (seconds)**	**LT_90_^d^ 95% C.L (seconds)**	**X^2^ (df)**	**P-value**	**Y= a+bx**
**DDT 4%**	−3.2516	1.2304±0.412	−	−	4.700 (3)	P< 0.05	Y= −3.2516+ 1.2304X
			439.28	4834.64			
			−	−			
**Permethrin 0.75%**	−2.5612	1.2573±0.182	76.00	667.98	5.524 (4)	P> 0.05	Y= −2.5612+ 1.2573X
			108.90	1138.58			
			148.41	2787.51			
**Deltamethrin 0.1%**	−1.7559	0.8823±0.297	0.02	444.34	18.26 (4)	P< 0.05	Y= −1.7559 +0.8823X
			97.75	2771.37			
			1022.94	−			
**Cyfluthrin 0.15%**	−0.3278	0.4686±0.105	0.50	608.75	1.73 (5)	P> 0.05	Y= −0.3278+ 0.4686 X
			5.00	2720.51			
			16.08	80988.38			
**Lambdacyhalothrin 0.05%**	−1.7522	0.9943±0.198	30.78	422.65	0.213 (2)	P> 0.05	Y= −1.7522+ 0.9943 X
			57.84	1125.10			
			109.68	8755.23			

a A= Intercept

b B±SE= Slope and its Standard Error

c LT_50_, 95% C.L= Leathal time cause 50% mortality and its 95% Confidence Limits

d LT_90_, 95% C.I= Leathal time cause 90% mortality and its 95% Confidence Limits

## Discussion

Due to the lack of a suitable guide line for sand flies, we had to use protocols of WHO for mosquito (19) in this study, the WHO exposure kit bioassay is widely accepted because it can measure insecticide susceptibility in many species of insect vectors worldwide (21). The assays can be run with live insects collected in the field or with their progeny reared in the laboratory. To control sand flies, populations around the world have been exposed to the four main classes of insecticides; 1) organochlorines, 2) organophosphates, 3) carbamates, and 4) pyrethroids, via residual spraying, ultra-low volume spraying, insecticide-treated clothing, and insecticide-treated nets. These exposures are either intentional in directed vector control efforts or are inadvertent as part of vector control efforts targeted against other insect vectors (22). Some sand fly populations have been found to be tolerant or resistant to the insecticides used in the Middle East, Southern Asia, and South America. In Montes Claros, Brazil, 29 of 80 (36.3%) *Lu. longipalpis* (Lutz and Nieva) survived a 0.05% deltamethrin exposure (23). Hassan et al. (2012) in the Surogia village of Khartoum State, Sudan, Reared sand flies in laboratory and they tested with malathion and propoxur. Results showed that 51 *P. papatasi* (79.7%) had insensitive acetylcholinesterase, which is associated with malathion and propoxur resistance. Both of these insecticides have been extensively used in this region as part of the anti-malaria mosquito control program (11). Denlinger et al. (2015) results showed that both laboratory sand flies *L. longipalpis* and *P. papatasi* are susceptible to DDT (24). Similar results with our study have been found in insecticide-susceptible Italian sand flies reared in laboratory (*P. perniciosus* and *P. papatasi*), where the LT50 and LT90 for DDT were longer compared with permethrin and lambdacyhalothrin (14). Also, Saeidi et al. (2012 Also, Saeidi et al. (2013) found both insecticide-susceptible male and female *P. papatasi* field and laboratory population to have much longer LT50 and LT90 to DDT than to permethrin, deltamethrin, cyfluthrin, and lambda-cyhalothrin (6, 12). Denlinger results suggested that laboratory colonies of insecticide- susceptible sand flies were not very susceptible to DDT (24). Shirani-Bidabadi et al. (2017) found that male and female *P. papatasi* field population to exposure to insecticides had much longer LT _50_ and LT _90_ to DDT than to permethrin, deltamethrin, cyfluthrin, and lambda-cyhalothrin (20). Our results of the tests at different durations of exposure indicated that laboratory sand fly populations from Esfahan province were fully susceptible to pyrethroid insecticides used, whereas the early tolerance (Resistant Candidate) was detected to DDT 4% after 3600s (1h) contact in the population of Esfahan Province. For both males and females, the susceptibility levels to DDT4% were greater than to pyrethroids. The high LT50 level of this vector to DDT was attributed to the long term use of insecticides for malaria vector control in the region that was transmitted genetically to their progeny. According to the report of the branch of the Ministry of Jahad in Esfahan Province, several herbicides, fungicides and inesticides have been used for agriculture and veterinary pest control in the region, including Methalaxile, Carbaryl, Permethrin, Cypermethrin, Deltamethrin, Metasystox-R and Mancozeb. The susceptibility test of a laboratory strain of *P. papatasi* to DDT4% and pyrethroids in Badrood shows that males were more susceptible than females to all the insecticides tested at LT _50_ level. In comparing our results and the study by Saeidi et al. (2013), LT _50_ values of female and male to DDT 4% was greater than LT _50_ in this study, males were more susceptible to pyrethroids and the sand flies were more tolerant compared to our results. LT50 values in males and females in our study to DDT 4%, Permethrin 0.75%, and Cyfluthrin 0.15% were also smaller compared to their results, but LT50 values in males and females to Deltamethrin 0.1% and Lambda-cyhalothrin 0.05% in our study were greater than theirs (12). This difference was due to the presence of Deltamethrin and Lambda-cyhalothrin in agriculture fields in recent years in Matin-Abad.

## Conclusion

We observed a clear difference between the insecticide susceptibilities of the *P. papatasi* laboratory population that had been exposed to insecticides in varying times intervals, this difference in susceptibility varied with sex. The result presented in this study can serve as starting points for determining the susceptibility of laboratory-reared *P. papatasi*, for determining diagnostic times for other sand fly species of public health concern. Knowing if a population of sand fly is resistant or resistance candidate to an insecticide or insecticide class is critical because it allows control strategies to be effectively implemented while not exacerbating the prevalence of insecticide resistance or resistance candidate in distribution area of sand flies. According to presented results, the reared population of sand flies collected from a hyper-endemic region of Esfahan Province is still susceptible to pyrethroids and resistance candidateto DDT 4%.
